# The Transforming Health Care Systems Partnership – collaboration for improvement post-pandemic

**DOI:** 10.1093/eurpub/ckac129.011

**Published:** 2022-10-25

**Authors:** S Montante

**Affiliations:** Coordinating Team Transforming Health and Care Systems Partnership, Brussels, Belgium

## Abstract

Building on the experience and results of important European initiatives like the TO-REACH Action, the new European Partnership on Transforming Health and Care Systems aims to accelerate the sustainable transformation of European health systems. By means of multidisciplinary research with a focus on improving the uptake of innovative solutions by health system actors, including organisational, service, and overarching policy innovations, the partnership plans to achieve access to high quality, sustainable and innovative health care for European populations. This presentation will explore current and upcoming plans for the Partnership, as well as discuss some of the lessons learnt from past initiatives and how these, combined with the experiences from the pandemic, have contributed to shaping the new Partnership's agenda. Inputs from stakeholders in the audience will be collected to inform the Partnership's ongoing learning process.

A country perspective from Slovenia

Vesna-Kestrin Petric

Slovenian Ministry of Health, Ljubljana, Slovenia

Health system strengthening and transformation – Current priorities of the European Commission

Isabel de La Mata

DG SANTE, European Commission, Brussels, Belgium

